# Bioleaching of olivine and enstatite with formation of Mg-oxalate mediated by engineered *Gluconobacter oxydans*

**DOI:** 10.1038/s41598-026-63817-0

**Published:** 2026-08-05

**Authors:** Jacob D. Klug, Luke Plante, James L. Adair, Alia Almansoori, Joseph J. Lee, Stephanie Murillo Maikut, Buz Barstow, Esteban Gazel

**Affiliations:** 1 Department of Earth and Atmospheric Sciences, Cornell University, Ithaca, NY 14853 USA; 2 Department of Biological and Environmental Engineering, Cornell University, Ithaca, NY 14853 USA

**Keywords:** Carbon sequestration, Critical elements, Geochemistry, Genetic engineering, Ultramafic, Olivine, Carbon capture and storage, Biotechnology, Biogeochemistry

## Abstract

Carbon mineralization using ultramafic rocks is a promising approach for long-term carbon dioxide removal. Here, we investigate whether a genetically engineered strain of *Gluconobacter oxydans* (B58 ∆pstS, P112:mgdh) can simultaneously achieve carbon mineralization and bioleach critical elements. Olivine and enstatite were bioleached at low-temperature conditions (30 °C) and with 5% pulp density. Direct *G. oxydans*-mineral contact promotes Fe^2+^ oxidation and leads to higher leaching efficiency compared to leaching with a cell-free biolixiviant. Importantly, *G. oxydans* facilitates the precipitation of magnesium oxalate, a compound with twice the carbon storage capacity of magnesite. Oxalic acid was detected in the *G. oxydans*-produced biolixiviant, and solid-phase Mg-oxalate formed most efficiently at low pH. SEM and XRD analyses reveal extensive olivine dissolution and secondary coating by Mg-oxalate and amorphous silica, which may inhibit further leaching. Mass balance calculations show that *G. oxydans* leached up to 75% of the Mg hosted in the starting materials, while only 11% of leached Mg reacted to sequester carbon as Mg-oxalate after 15 days. The enhanced sequestration potential of Mg-oxalate combined with bioaccelerated critical element leaching to offset costs represents a promising opportunity for global carbon storage that is worthy of further investigation. (193/200)

**Supplementary Information:**

The online version contains supplementary material available at 10.1038/s41598-026-63817-0.

## Introduction

The Intergovernmental Panel on Climate Change (IPCC) estimates that 5–10 Gt of CO_2_ will need to be removed from the atmosphere annually by 2050 to prevent the most harmful effects of the ongoing rise in global temperatures^1^. This requires the deployment of Gt-scale CO_2_ removal (CDR) technologies to securely sequester it from the atmosphere. One promising CDR method is carbon mineralization of ultramafic rocks^2–7^. Ultramafic rocks (rich in ferromagnesian minerals, i.e., olivine and pyroxene) form in Earth’s mantle and are far from equilibrium when at the surface, and thus represent a vast store of chemical potential energy^8^. Natural weathering breaks down ultramafic minerals releasing alkaline cations (e.g., Mg^+ 2^, Ca^+ 2^)^9,10^ that react with CO_2_ to form carbonate minerals. Therefore, ultramafic rocks can be naturally carbonated and sequester all anthropogenic CO_2_ emissions, but it will take too long (tens to hundreds of thousands of years)^11^ to mitigate the damaging effects of rising global temperatures^12–14^.

Bodies of ultramafic rocks like the Samail Ophiolite in Oman can naturally sequester 10^3^ tons of CO_2_ km^− 3^ yr^− 1^ but the limiting step in carbon mineralization is the low dissolution rates^15,16^. Microbes provide a new pathway to explore as they enhance chemical weathering and reduce the energy input necessary to dissolve Mg^2+^ from ultramafic rocks^17–19^. Leaching with microbial biolixiviants is currently applied to extracting metals from sulfides (20% of Cu and 5% of Au of the current global extraction) and is considered to be more sustainable than many conventional mining approaches^20–22^. *Gluconobacter oxydans* has demonstrated promise for biomining^23–29^. When fed glucose, it produces a biolixiviant rich in organic acids that can leach rare earth elements from phosphates and dissolve silicate ultramafic minerals^23–29^. Recent work demonstrated that *G. oxydans* significantly outperformed two other mineral dissolving microbes *Sphingomonas desiccabilis*, *and Penicillium simplicissimum*^29^. Additionally, the biolixiviant produced by *G. oxydans* leaches 3.2x more Mg from dunite compared to pure gluconic acid. Furthermore, the genetics of *G. oxydans* are relatively well understood making it an excellent candidate for further genetic engineering and construction of mutants tailored specifically for dissolving ultramafic rocks^21,22,28,29^.

Ultramafic rocks also contain elevated concentrations of critical elements such as Ni and Co. The demand for these critical elements has increased dramatically recently as nations transition to clean energy technologies^30,31^. Further, the demand for Ni and Co is projected to rise by nearly 20 times by 2040 owing to their high importance to geothermal, hydrogen, electric vehicles, and battery technologies^32^. Thus, simultaneous carbon mineralization and leaching of critical elements via bio-accelerated dissolution of ultramafic rocks provides a remarkable opportunity to offset the cost of CDR. But, the fundamental reactions, effects on leaching efficiency, and physiochemical processes that occur when *G. oxydans* interacts with ultramafic minerals are still unknown.

**Fig. 1:**
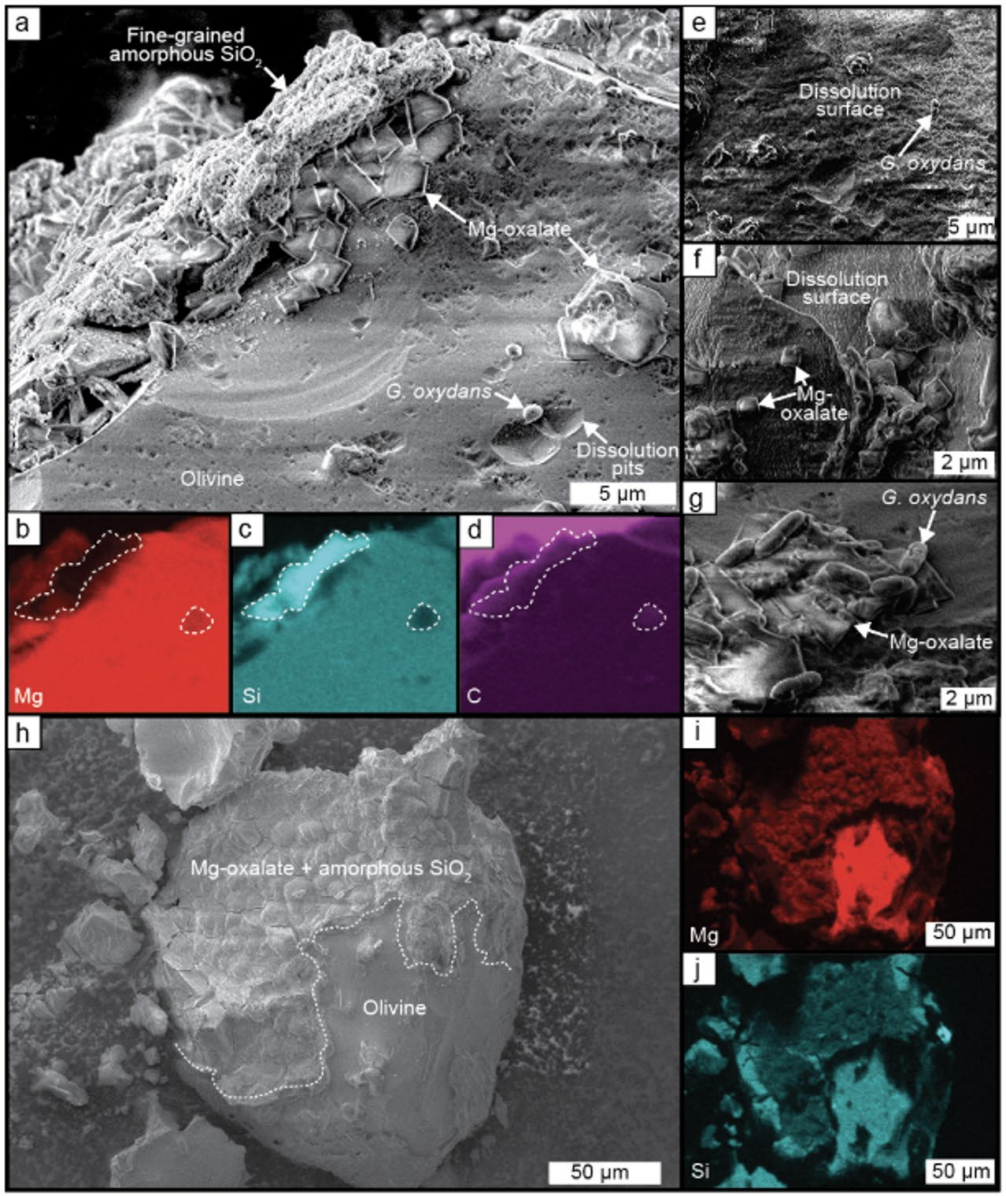
*G. oxydans* promotes olivine dissolution and precipitation of Mg-oxalate and amorphous SiO_2_. SEM-EDS images after 15 days of bioleaching demonstrating olivine dissolution textures, reaction products, and *G. oxydans.* (**a**) Tabular Mg-oxalate crystals, amorphous SiO_2_, and *G. oxydans* on the surface of a dissolution-pitted olivine (**b**–**d**) EDS Mg, Si, and C single element compositional maps of the image shown in (**a**), (**e**, **f**) Olivine surfaces with abundant dissolution pits and etch channels, (**g**) cluster of Mg-oxalate crystals with *G. oxydans* on olivine surface, (**h**) surface passivation example with Mg-oxalate and amorphous SiO_2_ reaction products almost completely covering the surface of olivine, (**i**, **j**) EDS Mg and Si single element compositional maps of the image shown in (**h**).

Here we investigate the bioleaching potential of *G. oxydans* (B58 *∆pstS*, P112:*mgdh*) on pure olivine and an olivine-pyroxene (enstatite) mixture as a proxy for ultramafic rocks. We tested both direct *G. oxydans* contact and the use of a biolixiviant-only approach where the bacteria are removed post-culture. Pure olivine and olivine-pyroxene powders were leached with the biolixiviant (± *G. oxydans*) at room temperature for 15 days with pH read and samples collected at days 0, 5, 10, and 15. Leachate solution chemistry was measured via inductively coupled plasma mass spectrometry (ICP-MS). The composition and textures of the solid residues were characterized with powder X-ray diffraction (XRD), scanning electron microscopy and energy dispersive spectroscopy (SEM-EDS), and Raman spectroscopy. Our results demonstrate that *G. oxydans* extracts up to 75% of the Mg hosted in the olivine starting material (quantified by mass balance based on starting composition, leachate ICP-MS, and XRD of post experiment solids). Thus, *G. oxydans* shows promise for accelerating the leaching of Mg and critical elements from ultramafic rocks. Following dissolution, we observed the precipitation of Mg-oxalate (Fig. 1a; also known as the mineral glushinskite [MgC_2_O_4_•2H_2_O])^33^. Mg-oxalate is a carbon-sequestering mineral that forms under room temperature and low pH conditions providing a promising pathway for carbon sequestration.

## Results

### Evolution of experiment leachate pH, Mg, and Ni concentrations

**Fig. 2:**
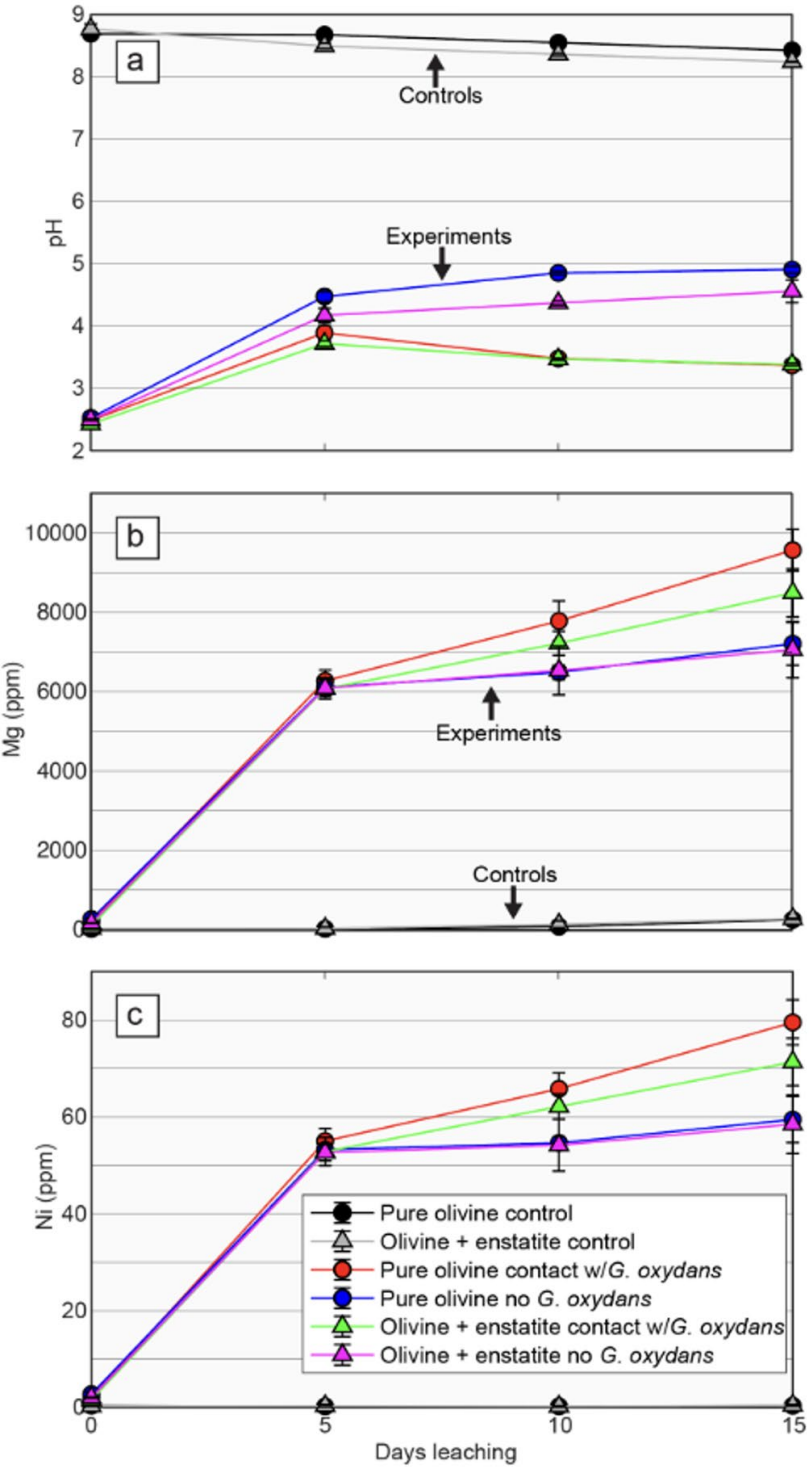
*G. oxydans* leaches Mg and Ni significantly more effectively than control experiments. Plots of (**a**) pH, (**b**) Mg concentration in the leachate, and (**c**) Ni concentration in leachate on each sample day of the bioleaching experiments. Error bars are ± 1σ.

Leachate pH was recorded on experiment days 0, 5, 10, and 15 (Fig. 2a). The pH was lowest at the beginning of the experiments with all samples having a pH of ~ 2.5. By day 5 the pH of all non-control experiments rose to > 3.7. However, leachates from biolixiviant-only experiments increased to a higher pH than when *G. oxydans* was present. Following Day 5, the pH values of the leachates with *G. oxydans* versus biolixiviant-only continued to diverge (Fig. 2a), with the pH of the *G. oxydans* bearing leachates dropping to 3.4 while the *G. oxydans*-free leachates pH increased to 4.9 (pure olivine) and 4.6 (olivine-enstatite).

The largest amount of Mg and Ni is leached in experiments where *G. oxydans* was in direct contact with olivine and enstatite, these experiments also had the lowest pH values (Fig. 2a-c). By Day 5, leachate Mg and Ni concentrations increased sharply in all experiments to greater than 6000 ppm and 50 ppm, respectively. Following day 5, leaching experiments with *G. oxydans* performed better than biolixiviant-only experiments (Fig. 2b-c). The pure olivine + *G. oxydans* experiments were the top performers; on average it leached more than 9500 ppm Mg and 80 ppm Ni. For the *G. oxydans*-free experiments, the amount of Ni and Mg leached increased by only ~ 6 ppm Ni and 1000 ppm Mg following day 5. Less than 280 ppm Mg and 0.5 ppm Ni was leached in all the control samples.

### Characterization of solid experimental residues

**Fig. 3:**
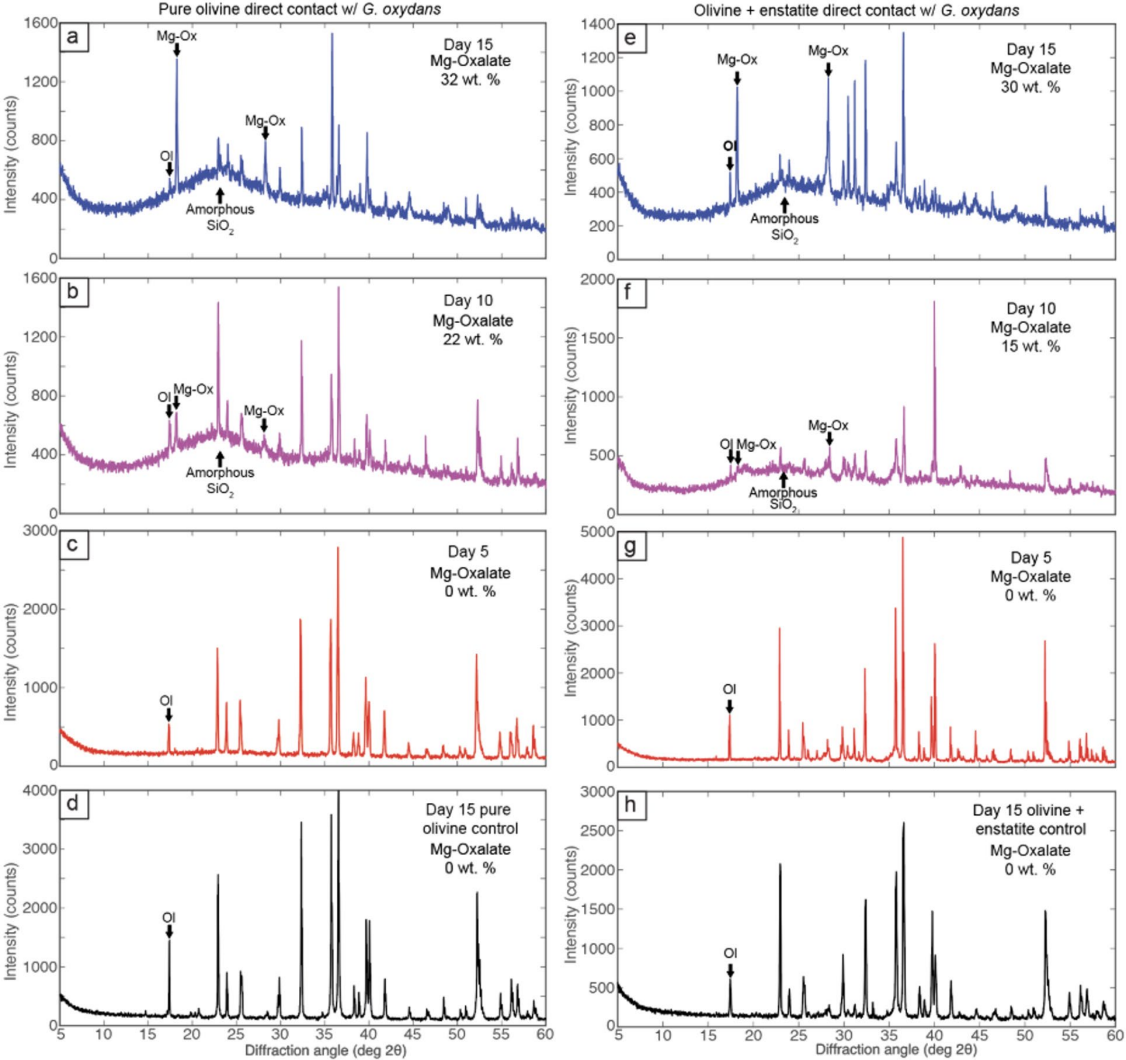
Mg-oxalate and amorphous SiO_2_ XRD peaks grow as forsterite peaks diminish due to dissolution. Compilation of XRD spectra from experiments where *G. oxydans* was left in contact with the ultramafic powders. (**a**–**c** & **e**–**g**) Show progressive leaching on day 5, 10, and 15, while (**d**–**h**) spectra on the bottom are day 15 control samples. The spectra on the left show pure olivine leaching experiments and those on the right show olivine + enstatite. Arrows point to olivine and Mg-oxalate peak positions which diminish and increase in intensity, respectively, throughout leaching. Also, note the appearance of broad peak on day 10 and 15 indicative of amorphous SiO_2_ (**a**, **b**, **e**, & **f**).

XRD analysis of post-experimental solid residues shows formation of amorphous SiO_2_ and Mg-oxalate throughout leaching (Fig. 3). The presence of amorphous SiO_2_ is indicated by a broad peak spanning 15 to 30º 2θ (e.g., ref^34^). By day 15, the formation of amorphous SiO_2_ is observed in all non-control experiments with the broad peak also being most intense on day 15. Similarly, Mg-oxalate peaks are most intense in the day 15 leaching experimental residues. Mg-oxalate is not observed to form until day 10, after which the amount increases progressively with day 15 residues containing the most (Fig. 3a & e). No amorphous SiO_2_ or Mg-oxalate peaks are observed in any control experiments (Fig. 3d & h), this precludes formation of Mg-oxalate or amorphous SiO_2_ because of pre-experiment autoclaving.

**Fig. 4:**
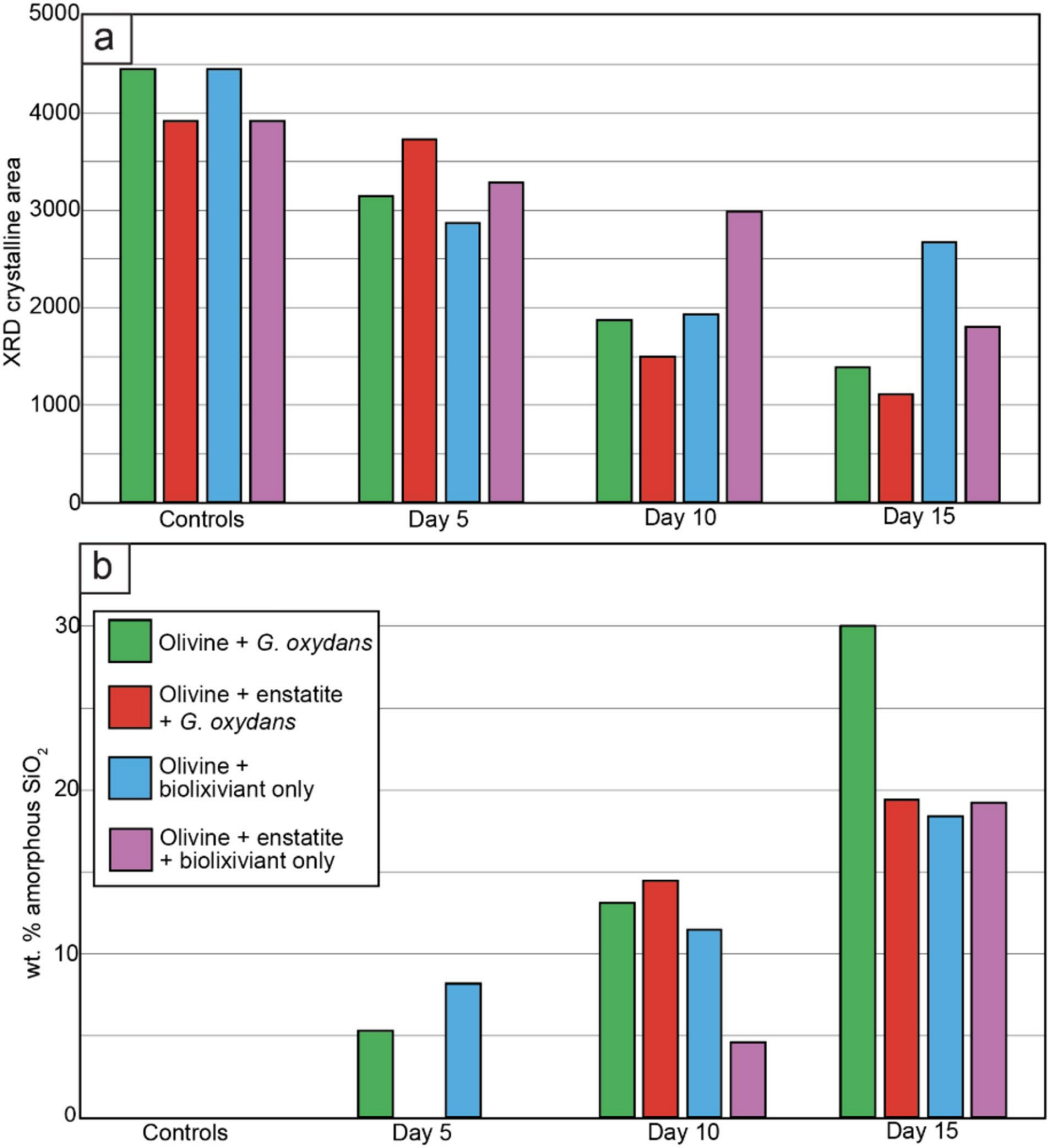
Olivine and enstatite dissolves as amorphous SiO_2_ precipitates. (**a**) Total crystalline area calculated from area under peaks in XRD spectra. (**b**) Plot of wt% amorphous SiO_2_ calculates via degree of crystallinity from XRD spectra^35,36^. Degree of crystallinity method has an uncertainty of < 5 wt% for determining amorphous SiO_2_ totals. Biolixiviant only samples do not contain *G. oxydans*.

Correspondingly, olivine peaks decrease in intensity throughout the experiments, indicating olivine is being dissolved throughout leaching (Fig. 3). The total crystalline area beneath peaks from the XRD spectra drops progressively from day 0 to 15 as olivine dissolves (Fig. 4a). The leaching experiments with *G. oxydans* were most effective at forming Mg-oxalate, with the olivine-enstatite and pure olivine day 15 solid residues containing 30 and 32 wt%, respectively. Mg-oxalate is also observed in the pure olivine biolixiviant-only leaching experiments but is less abundant than when *G. oxydans* is present (20 versus 32 wt%). XRD spectra from the olivine-enstatite biolixiviant-only experiments do not indicate Mg-oxalate formation. The amount of amorphous SiO_2_, determined using the degree of crystallinity method^35,36^, also increased throughout the leaching experiments (Fig. 4b). The experiments with pure olivine + *G. oxydans* formed the most amorphous SiO_2_ (30 wt%) by day 15.

The formation of secondary phases was also confirmed with SEM imaging and EDS compositional mapping (Fig. 1b-d). The amorphous SiO_2_ formed as nanometer-sized particles clumped on the surface of olivine crystals often directly associated with Mg-oxalate. Mg-oxalate is observed as tabular crystals typically 2–10 μm in size. Consistent with the composition of Mg-oxalate, EDS mapping showed that the oxalate crystals lacked silicon and were enriched in carbon relative to adjacent olivine (Fig. 1a, c-d, h-j). The presence of Mg-oxalate was also confirmed via Raman spectroscopy (Fig. S1) with peak positions consistent with those outlined for natural Mg-oxalates^37^.

### Biolixiviant compositional analysis

Gas chromatography-mass spectrometry (GC-MS) analysis confirmed the presence of gluconic acid, in addition to several isomers including mannonic acid, altronic acid, and allonic acid (Supplementary data). Notably, oxalic acid was also detected in a mutant sample. Preliminary calibrated high performance liquid chromatography (HPLC) analysis showed wild type (non-engineered) biolixiviant to be approximately 6% gluconic acid, with the B58 ∆*pstS*, P112:*mgdh* mutant (genetically engineered) biolixiviant containing about 10% gluconic acid. Thus, the mutant B58 ∆*pstS*, P112:*mgdh* used in these experiments produces a more acid-rich biolixiviant than the wild type *G. oxydans*.

## Discussion

### Olivine dissolution and the formation of Mg-oxalate

*G. oxydans* has previously been demonstrated to produce a biolixiviant that is predominantly composed of gluconic acid ^38,39^. Yet, the presence of Mg-oxalate in the solid experimental residues indicates the formation of oxalate (C_2_O_4_^2−^) in the biolixiviant. Indeed, oxalic acid was present in one of the mutant biolixiviant replicates analyzed via GC-MS, though more work is needed to understand the proportions of organic acids present in the biolixiviant. Biosynthesis of oxalic acid by microbes, especially fungi, is a common process^40^. Specifically, precipitation of Mg-oxalate has been documented previously with the *Aspergillus niger* fungi^41,42^. There are metabolic pathways that convert glucose into oxalic acid consuming CO_2_^43^, in these scenarios if Mg-oxalate is stable its formation will lead to the sequestration of carbon. Oxalic acid is diprotic such that its speciation is pH dependent^44^, as outlined by the dissociation reactions below^44^:

1 $${\mathrm{H}}_{{\mathrm{2}}} {\mathrm{C}}_{{\mathrm{2}}} {\mathrm{O}}_{{\mathrm{4}}} \leftrightarrow {\mathrm{HC}}_{{\mathrm{2}}} {\mathrm{O}}_{{\mathrm{4}}} ^{ - } + {\text{ H}}^{ + } \left( {{\mathrm{H}}_{{\mathrm{2}}} {\mathrm{C}}_{{\mathrm{2}}} {\mathrm{O}}_{{\mathrm{4}}} {\text{dominant below pH 1}}.{\mathrm{25}}} \right)$$

2 $${\mathrm{HC}}_{{\mathrm{2}}} {\mathrm{O}}_{{\mathrm{4}}} ^{ - } \leftrightarrow {\mathrm{C}}_{{\mathrm{2}}} {\mathrm{O}}_{{\mathrm{4}}} ^{{{\mathrm{2}} - }} + {\text{ H}}^{ + } \left( {{\mathrm{C}}_{{\mathrm{2}}} {\mathrm{O}}_{{\mathrm{4}}} ^{{{\mathrm{2}} - }} {\text{dominant above pH 4}}.{\mathrm{26}}} \right)$$

During olivine dissolution experiments ref^44^ observed less oxalate precipitation at higher concentrations of oxalic acid which they attributed to a lack of free oxalate ions (C_2_O_4_^2−^) at lower pH (Eqs. 1 and 2). However, we observe the least Mg-oxalate precipitation in the experiments without *G. oxydans* where the pH was higher (Fig. 2a) and free oxalate ions should have been the dominant species. Instead, Mg-oxalate formed most efficiently in the lower pH experiments when *G. oxydans* remained in contact with the rock powder and HC_2_O_4_^−^ should have been the dominant oxalate species (Eqs. 1 and 2). This may indicate that *G. oxydans* itself promotes the precipitation of Mg-oxalate.

Dissolution pits and etching channels are abundant on olivine residue surfaces and are consistent with extensive leaching (Fig. 1a, e-f). Further evidence of dissolution is demonstrated by the XRD spectra which show the total crystalline area generally decreasing and amorphous SiO_2_ increasing as bioleaching progresses (Fig. 4). Ref^45^ showed that dissolution of olivine in oxalic acid is pH dependent where H_4_SiO_4_ is released into solution in a rate-limiting protonation step, and that Mg dissolution is likely incongruent with other elements. This is consistent with our leaching experiments, which demonstrate that more Mg and Ni were leached from the experiments that had the lowest average pH values (Fig. 2a). Simplified reactions to show olivine dissolution (Eq. 3) and the formation of Mg-oxalate and amorphous SiO_2_ (Eq. 4) are given below:

3 $$\left( {{\mathrm{Mg}},{\mathrm{Fe}}} \right)_{{\mathrm{2}}} {\mathrm{SiO}}_{{\mathrm{4}}} + {\mathrm{2HC}}_{{\mathrm{2}}} {\mathrm{O}}_{{\mathrm{4}}} ^{ - } + {\mathrm{2H}}_{{\mathrm{2}}} {\mathrm{O}} + {\mathrm{2H}}^{ + } ~ \to {\mathrm{Mg}}^{{{\mathrm{2}} + }} + {\mathrm{Fe}}^{{{\mathrm{2}} + }} + {\mathrm{H}}_{{\mathrm{4}}} {\mathrm{SiO}}_{{\mathrm{4}}} + {\mathrm{2C}}_{{\mathrm{2}}} {\mathrm{O}}_{{\mathrm{4}}} ^{{{\mathrm{2}} - }} + {\mathrm{2H}}_{{\mathrm{2}}} {\mathrm{O}}$$

4 $$\begin{gathered} {\mathrm{Mg}}^{{{\mathrm{2}} + }} + {\mathrm{Fe}}^{{{\mathrm{2}} + }} + {\mathrm{H}}_{{\mathrm{4}}} {\mathrm{SiO}}_{{\mathrm{4}}} + {\mathrm{2C}}_{{\mathrm{2}}} {\mathrm{O}}_{{\mathrm{4}}} ^{{{\mathrm{2}} - }} + {\mathrm{1}}.{\text{5 H}}_{{\mathrm{2}}} {\mathrm{O}} + 0.{\mathrm{25O}}_{{\mathrm{2}}} \to {\mathrm{MgC}}_{{\mathrm{2}}} {\mathrm{O}}_{{\mathrm{4}}} \cdot {\mathrm{2H}}_{{\mathrm{2}}} {\mathrm{O}} + {\mathrm{SiO}}_{{\mathrm{2}}} \hfill \\ + {\mathrm{FeO}}\left( {{\mathrm{OH}}} \right) + {\mathrm{C}}_{{\mathrm{2}}} {\mathrm{O}}_{{\mathrm{4}}} ^{{{\mathrm{2}} - }} + {\mathrm{2H}}^{ + } \hfill \\ \end{gathered}$$

Clusters of Mg-oxalate and amorphous SiO_2_ are observed on the surface of olivine (Fig. 1a, f-h). In some instances, the layers of Mg-oxalate and amorphous SiO_2_ are observed to nearly completely cover the surface of olivine (Fig. 1h-j). The precipitated amorphous SiO_2_ and Mg-oxalate progressively covers the olivine surfaces as the bioleaching experiments progress. This surface passivation appears to reduce bioleaching efficiency over time as secondary products form a layer^46,47^ that shields olivine from the biolixiviant and slows dissolution rates. This is reflected in the ICP-MS data (Fig. 2b-c) that shows Mg and Ni leaching slows significantly after day 5.

### Direct contact with *Gluconobacter oxydans* accelerates bioleaching

**Fig. 5:**
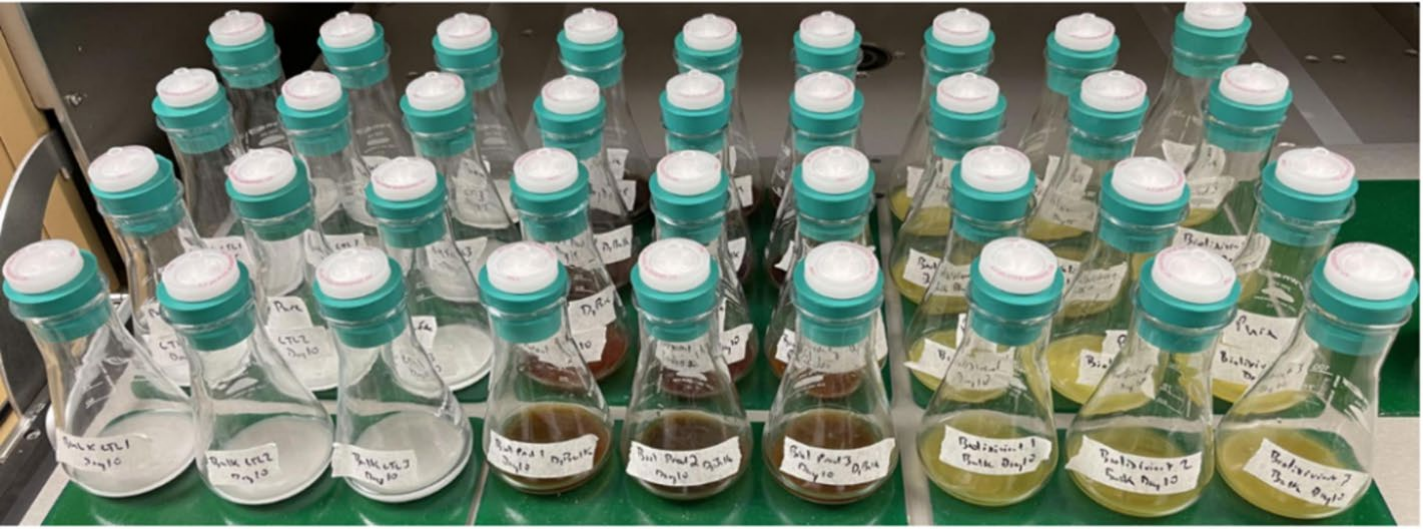
Contact with *G. oxydans* promotes oxidation of Fe^2+^. Image showing different leachate colors in bioleaching flasks, the flasks on the left are control experiments, those in the middle contain *G. oxydans*, and those on the right are biolixiviant only experiments. Notice that the *G. oxydans* containing flasks are brown suggesting Fe^2+^ to Fe^3+^ oxidation.

There are striking differences between the leaching experiments where *G. oxydans* was in direct contact with olivine compared to when *G. oxydans* was absent (Fig. 5). The experiments with *G. oxydans* evolved from white/clear to dark red/brown, while those without are light yellow (controls stayed white/clear all the time). This implies *G. oxydans* is interacting with and altering olivine in ways other than just producing biolixiviant that promotes dissolution. The red/brown color is consistent with a redox reaction converting Fe^2+^ (as found in the structure of olivine and pyroxenes) to Fe^3+^. This reaction has been observed in other biomining processes where microbes oxidize Fe^2+^ to Fe^3+^ while utilizing electrons as an energy source^48,49^ or to limit toxicity from Fe^2+^^50^. However, *G. oxydans* is not known to be able to oxidize Fe^2+^. Yet if it were purely abiotic oxidation, it would be expected that the dissolved Fe^2+^ in the experiments without *G. oxydans* would have also turned the leachate red/brown, but this is not observed. Thus, the presence of *G. oxydans* is at least indirectly leading to the oxidation of Fe^2+^. One potential mechanism is that *G. oxydans* is generating reactive oxygen species (e.g., O_2_^−^, H_2_O_2_) that then react with dissolved Fe^2+^ oxidizing it to Fe^3+^. A similar pathway has been observed in other aerobic bacteria (*Arthobacter oxydans)* that oxidize Mn to reduce toxicity^51^.

This is the first observation that *G. oxydans* mutants may be able to oxidize iron or otherwise promote the oxidation of iron. Olivine dissolution rates are controlled primarily by pH, water activity, temperature, and mineral-fluid surface area^52^. Thus, if *G. oxydans* can oxidize iron to extend its lifespan, allowing it to produce additional biolixiviant and keep pH lower, then it could aid substantially in accelerating bioleaching of ultramafic rocks. The olivine + *G. oxydans* leaching experiments had the lowest pH values (Fig. 2a) which may suggest that *G. oxydans* can tolerate the release of metals and surge of alkalinity that occurs during ultramafic rock bioleaching, thus allowing it to survive and continue producing acid. Additionally, the amount of initial Fe in solution by day 15 of leaching is ~ 3–5% less than Mg (Supplementary data). This may indicate that secondary Fe-oxide minerals (e.g., goethite) are forming leading to lower amounts of Fe in solution relative to Mg. The presence of Fe-oxide minerals may contribute to the color change observed in the *G. oxydans* bearing experiments. However, it is more likely the red/brown solution (Fig. 5) produced is caused by primarily by solubilization of Fe^3+^. This may be through chelation via metabolites (likely siderophores) released by *G. oxydans* or potentially complexation with oxalate to form soluble ferric-oxalate complexes^53,54^. Siderophores, which have an extremely high affinity for binding iron, have been shown to increase olivine dissolution via the liberation of surface-bound Fe^19^. Endogenous siderophores expressed in *G. oxydans* B58 are potentially involved in the color change due to the increased iron in solution. In either scenario, these results indicate that the physical presence of *G. oxydans* encourages oxidation of Fe^2+^ and play an integral role in promoting dissolution. This may preclude the production of a synthetic lixiviant that performs as well as the biolixiviant with *G. oxydans*. However, more work is needed to better understand how *G. oxydans* interacts with iron derived from ultramafic rocks. Future work on a detailed characterization of all chemical species present in the biolixiviant (organic acids and metabolites) before and after exposure to ultramafic minerals would deepen our understanding of the processes occurring during bioleaching. Additionally, a future genetic screen investigating biologically synthesized proteins and metabolites would also reveal critical biological components of the biolixiviant that contribute to olivine dissolution. Further, pairing these biological characterizations with metal-centric speciation analyses (i.e., XANES) would be a valuable next step toward a more complete picture of Fe, Mg, and Ni behavior in this system.

### Evolution of Mg distribution throughout leaching

**Fig. 6:**
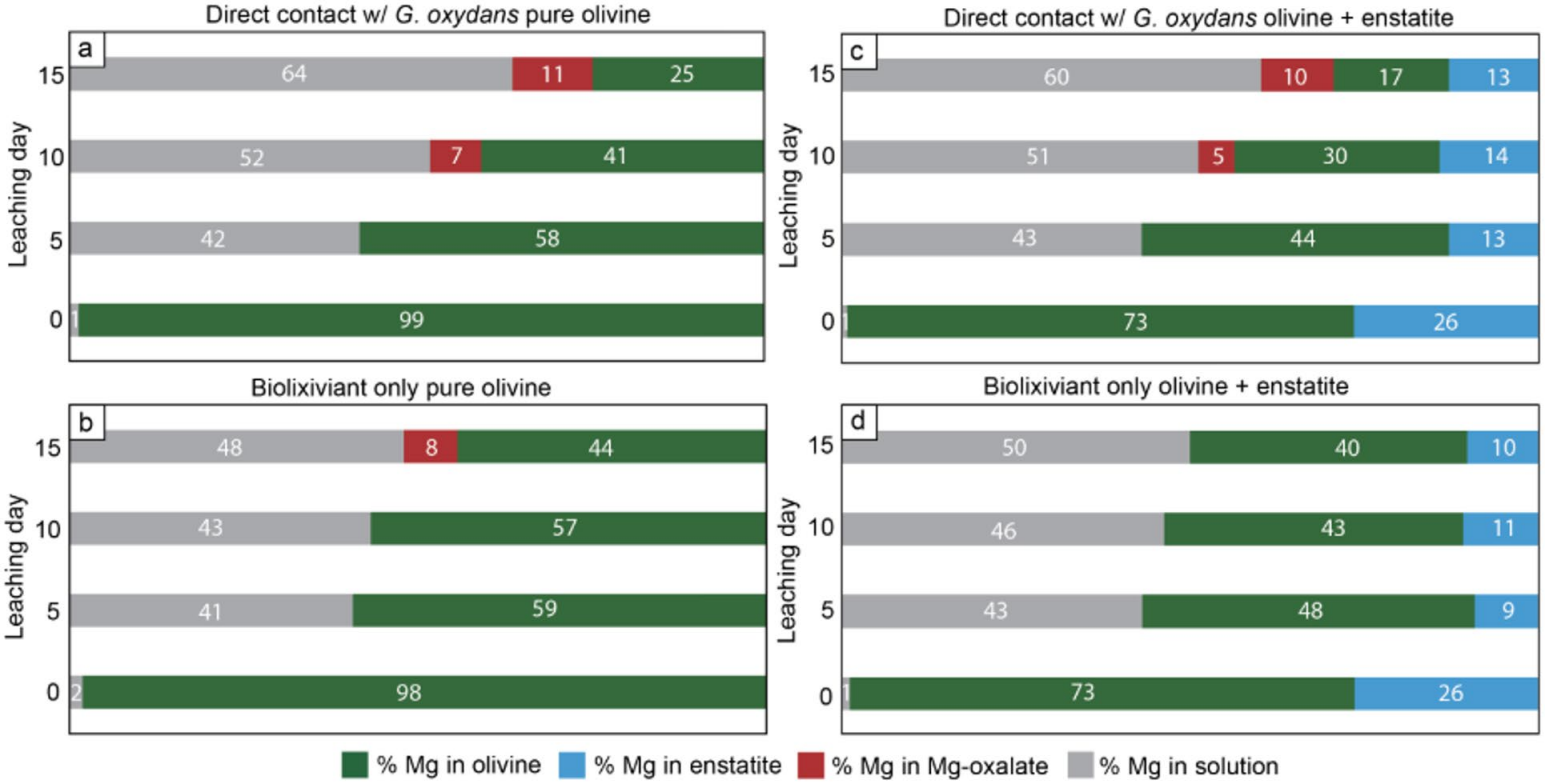
Mg mass balance calculations show up to 75% of the original Mg dissolved and up to 11% has been incorporated into Mg-oxalate. Extent of progressive dissolution and carbon mineralization demonstrated with the proportion of total Mg in olivine, enstatite, Mg-oxalate, and solution on bioleaching day 0, 5, 10, and 15. (**a**, **b**) show pure olivine experiments, while (**c**, **d**) show olivine-enstatite experiments. Panels (**a**, **c**) show experiments with the most successful dissolution where *G. oxydans* was left in, while panels (**b**, **d**) are *G. oxydans*-free experiments and show less efficient dissolution.

Mass balance calculations were performed to track where Mg is distributed throughout the leaching experiments which serves as a useful proxy for dissolution progress and rates of carbon mineralization. For these calculations we assumed a forsterite composition of Mg_1.81_Fe_0.19_SiO_4_ (Table S1) and an enstatite composition of MgSiO_3_. The amount of Mg in solution is calculated using the concentrations from ICP-MS analyses and 10 mL of solution. The amount of Mg measured in solution (via ICP-MS) is then subtracted from the starting Mg to determine how much Mg remains in the solid residue. The proportions of minerals in the solid residues (from XRD) were used to calculate how much Mg is present in each of the mineral phases (olivine, enstatite, and Mg-oxalate) at each stage of leaching (Days 0, 5, 10, and 15). By day 15, at least 50% of the total Mg is leached from the starting material in all experiments (Fig. 6). By day 15, at least 70% of all initial Mg was leached in the experiments where *G. oxydans* was present. For comparison, this is significantly higher Mg extraction efficiency than that reported for the fungus *Aspergillus niger*. In an olivine bioleaching experiment conducted at the same pulp density, *A. niger* extracted only ~ 10% of the Mg over 21 days^55^. Magnesium appears to be more efficiently leached from olivine compared to enstatite. For example, only about half of the initial Mg hosted in enstatite is liberated by day 15 of the olivine-enstatite + *G. oxydans* experiments, compared to 75% of the initial Mg in the pure olivine + *G. oxydans* experiments (Fig. 6a&c).

Additional mass balance calculations show that *G. oxydans* preferentially leached Mg relative to other elements like Fe and Ni (Supplementary data). By day 15, the average proportion of the total original Fe and Ni that dissolved in solution is 4% and 12% lower, respectively, than the proportion of the original Mg that was leached. These values may indicate incongruent dissolution of olivine in oxalic acid (e.g., ref^45^). Alternatively, these differences could reflect incorporation of Fe and Ni into secondary phases (oxalates, Fe-oxides) that is not accounted for in the calculations.

### Carbon sequestration potential with Mg-oxalate

Mg-oxalate presents a valuable opportunity for carbon mineralization since it can bond with two carbons per magnesium versus a 1:1 ratio for magnesite (MgCO_3_). For example, there are ~ 10 Gt of ultramafic mine tailings in existence^4^. Ultramafic mine tailings already have a reduced grain size and would be a natural starting point for bio-accelerated carbon mineralization projects. If all the Mg in the currently available mine tailings reacted to form magnesite approximately 1.2 Gt of C would be captured, versus 2.4 Gt of C if the Mg reacted to form Mg-oxalate (assuming the tailings contain 41 wt% MgO). Further, Mg-oxalate can form at room temperature and low pH immediately following dissolution which could be an advantage since it may not require energy inputs for heating or an additional step to raise pH as is often needed to form magnesite^44^. However, the carbon that is captured by the Mg-oxalate in our experiments likely ultimately is sourced from the glucose feedstock, where the oxalate (C_2_O_4_^−2^) is generated from the breakdown of gluconic acid. Thus, the amount of oxalic acid (and subsequent Mg-oxalate) that can be produced is limited by how much gluconic acid is synthesized by *G. oxydans*. Further, for this process to help mitigate rising atmospheric CO_2_ concentrations, alternative feedstocks that would otherwise re-emit their carbon to the atmosphere will need to be utilized. The high cost of glucose as a feedstock for microbial activity already serves as a barrier to scalability for biomining and carbon sequestration with *G. oxydans.* Thus, alternative, more economical options such as cellulosic hydrolysate are being investigated to make large-scale applications feasible^28,29^.

In the pure olivine + *G. oxydans* experiments done here only 11% of the total Mg had been incorporated into Mg-oxalate by day 15 of leaching. Thus, 64% of the original Mg remained dissolved in solution (Fig. 6a), available to react to form carbon sequestering minerals. Mg-oxalate is not observed until day 10 in any of the experiments, so perhaps longer duration experiments would lead to a greater extent of oxalate formation. Additional work is needed to develop procedures that promote and optimize the crystallization of Mg-oxalate if it is to be utilized for carbon mineralization. While much of the Mg is in solution following leaching, its availability to form Mg-oxalate depends on its molecular speciation, thus characterizing the dissolved Mg speciation (e.g., via ESI-MS and geochemical modeling) is an important direction for future work. Furthermore, dissolution is impacted by surface passivation. Mg-oxalate and amorphous silica coatings progressively cover olivine surfaces (Fig. 1h-j) shielding it and limiting further dissolution. This caps the total Mg that can be liberated and, thus, the carbon that can be stored. Strategies to renew the reacting surface will likely be needed to achieve optimal carbon storage.

Furthermore, questions remain regarding the stability of Mg-oxalate and its suitability as an option for permanent carbon storage. Mg-oxalate dihydrate (glushinskite) is a rarely occurring mineral in nature^33^, thus there has been limited work to understand how it forms, weathers, and its environmental implications^42,44^. Compared to magnesite, Mg-oxalate is more susceptible to chemical weathering, it is approximately twice as soluble in H_2_O (Mg-oxalate 0.038 g/100 g H_2_O versus magnesite 0.018 g/100 g H_2_O)^56^. Thermally, Mg-oxalate should be stable at surface conditions, water is liberated from Mg-oxalate when heated to 110–170 °C, but its carbon will not be released until the oxalate component decomposes at 270–397 °C^57,58^. This higher temperature thermal decomposition leaves behind nanocrystalline MgO which potentially has industrial uses for catalysis, catalyst supports, toxic waste remediation, refractory materials, and adsorbents^59^. Thus, Mg-oxalate generated via biomining could also be a starting material for these industrial applications. Using commercially available synthetic oxalic acid to dissolve olivine and precipitate Mg-oxalate would be prohibitively expensive, costing more than $1000 USD per ton of carbon^44^. Cost-effective microbial production of oxalic acid is an area of ongoing research^60^ and warrants further investigation with *G. oxydans*.

## Methods

### San Carlos ultramafic starting materials and characterization

The forsterite-rich San Carlos olivine is well studied and often used as a starting material for experiments^61–64^ and has been used as an electron microprobe reference material (USNM 111312/444^65^). Commercially acquired San Carlos has been demonstrated to be more variable in both major and trace element composition than USNM San Carlos olivine^66,67^. An average composition of commercially acquired San Carlos Olivine based on measurements from ref^67^ is shown in Table S1. A bulk sample of ultramafic minerals from the San Carlos Apache reservation in Arizona was acquired commercially^68^. The bulk material consists primarily of olivine (Fig. S2), while containing some enstatite, and trace amounts of clinopyroxene. Some grains had a coating of carbonate on their exterior, these crystals were removed. Following this, the remaining grains were washed in hydrosonic bath with deionized water for 60 min to remove any remaining carbonate adhering to crystals. The remaining grains were inspected a second time and any grains with carbonate or evidence of alteration were removed. Following this, a split of the bulk material (olivine + enstatite) was set aside, all enstatite and clinopyroxene grains were then removed from the remaining split leaving a pure sample of San Carlos olivine (Fig. S2). The olivine + enstatite and pure olivine samples were crushed for 20 min in a tungsten carbide vial to create a fine-grained powder (typical particle size = 40 μm) using a SPEX SamplePrep 8000 M mill. The powder was thoroughly mixed and homogenized. The mineralogy of the powders before and after exposure to a biolixiviant was analyzed via x-ray diffraction using a Bruker D8 Advance ECO powder diffractometer at Cornell Center for Materials Research (CCMR). The pure sample was confirmed to initially contain 100% olivine, while the bulk sample initially contained 69 wt% olivine, and 31 wt% enstatite (Fig. S2). Measurements were performed at 5 to 60º 2θ, with a step size of 0.019º and a dwell time of 0.3 s per step. To reduce the effect of Fe fluorescence the detector’s lower discriminator was elevated from 0.110 V to 0.182 V.

### G. oxydans and leaching experiment conditions

*Gluconobacter oxydans* B58 *∆pstS*, P112:*mgdh* (the best performing mutant engineered in the authors’ laboratory in previous work^23^) was the strain used throughout these tests. The mutant was produced in the laboratory previously^23^ and stored at -80℃ in 30% glycerol v/v. Technical replicates were used for these experiments: a single colony from a yeast extract, peptone, and mannitol (YPM) plate was selected, grown in 25 mL of liquid YPM^69^, and split into all flasks. This resulted in a single colony used among all experiments.

A YPM plate^69^ of this mutant was grown for two days at 30℃. A single Erlenmeyer flask (all Erlenmeyer flasks used were VWR 100 mL Cat. No. 75804-644) was filled with 25 mL of YPM, inoculated with a single colony of the *G. oxydans* mutant, and grown for two days at 30℃ and at 200 rpm. This single liquid culture then inoculated at 0.05 optical density (OD; read at 590 nm using a WPA Colourwave CO7000) each of the 48 separate 100 mL flasks with YPM for a total volume of 20 mL of YPM plus inoculation culture per flask.

After the two days of growth, flasks were decreased in volume to 10 mL of liquid culture, and 10 mL of 40% w/v glucose (filtered using a 0.22 µm filter) were added to each flask for a total volume of 20 mL per flask. These samples were then set at 30 ℃ and 200 rpm for two more days for biolixiviant production. Mineral powders were sterilized at 135℃ for three minutes at 30 psi above atmospheric pressure. Next, the samples where *G. oxydans* was left in were decreased in volume to 10 mL each to increase headspace and decrease culture depth to facilitate oxygen transfer to the aerobic *G. oxydans.* Next, 5% pulp density (0.5 g) of powdered olivine ± enstatite was added to the direct leaching samples. The remaining samples were then centrifuged and filtered using 0.2 µm filters to remove the *G. oxydans* from the samples. New flasks were then filled with 10 mL of filtered biolixiviant for the indirect leaching samples, and 0.5 g of olivine ± enstatite was added. All flasks were covered with No. 7 neoprene stoppers bored with a 3/16” drill bit, and hydrophobic filters were inserted into the holes bored into the filters. These filters allowed for gas exchange while preventing evaporation from the flasks. Triplicate experiments were conducted for each sample day 0, 5, 10, and 15, for different starting mineral types and leaching methods. There were, therefore, 12 biological flasks per sample day: three for leaching of pure olivine with biolixiviant + *G. oxydans*, three for leaching of pure olivine with biolixiviant only, three for leaching of enstatite and olivine with biolixiviant + *G. oxydans*, and three for leaching of enstatite + olivine with biolixiviant only. The flasks were then set at 30 ℃ with no mixing for the corresponding leaching times. Controls consisted of flasks filled with 10 mL of 20% glucose and 0.5 g of olivine ± enstatite. The controls were intentionally free of any *G. oxydans*, biolixiviant, or other organic acids to adjust pH. To investigate the fundamental reactions, leaching efficiency, and physiochemical processes that occur due to *G. oxydans* and its biolixiviant, it was necessary to include control experiments in which both *G. oxydans* and the acidic biolixiviant were absent, allowing microbial effects to be distinguished from any background interactions between the minerals and the experimental medium.

Upon collecting samples, the flask contents were centrifuged for 20 min at 3,214 × *g*, 1.5 mL of centrifuged leachate was saved in 1.7 mL centrifuge tubes at -80℃ for future analysis in an ICP-MS, and pHs were taken using a VWR Symphony B10P. The remaining leachate was discarded, and the leached powdered rocks were washed with deionized (DI) water, centrifuged again for 20 min at 3,214 × *g*, and dried at room temperature for future analysis.

### Post-experimental characterization

ICP-MS measurements were completed using the routine of ref^29^. Frozen samples were thawed and centrifuged using 0.45 μm polypropylene 96-well filter plates into polypropylene 96-well plates for 10 min at 3,214 × g. The resulting samples were then diluted to 0.01% (a 10,000x dilution) of their original concentration in 5 mL of a 2% nitric acid solution. Subsequently, the samples were analyzed with an Agilent 7800 ICP-MS at the Cornel Mass Spectrometry Facilities at the Department of Earth and Atmospheric Sciences. The ICP-MS standard was a custom preparation by High Purity Standards, containing 1,000 µg/mL of iron, magnesium, and silicon, along with 100 µg/mL of calcium, cobalt, chromium, copper, potassium, manganese, sodium, nickel, phosphorus, titanium, and zinc. An internal standard mixture of Be, Ge, In, Li, Lu, Rh, Sc and Tb was used to correct for instrument drift and variability. A calibration curve was created using standards of known concentration. Blanks and replicates were analyzed alongside the samples to assess accuracy and precision respectively. Obtained data was processed using the software Mass Hunter 4.5, version C.01.05.

When the 10,000x diluted samples were analyzed via ICP-MS a consistent offset to higher concentrations was observed. For example, day 0 control samples were offset to higher concentrations by ~ 1000 ppm Mg and ~ 3 ppm Ni. Thus, a blank correction was performed by taking the average day 0 control concentration from the 10,000x dilution and subtracting from it the average day 0 control concentrations from a set of 100x dilution ICP-MS measurements on the same samples where the offset was not observed. Using this approach a blank correction factor of 1043 ppm Mg and 3.2 ppm Ni was determined. These values were subtracted from the 10,000x dilution leachates and controls, the results are plotted in Fig. 2b-c. The blank corrected data for all elements is given in the supplementary data file.

Following extraction of the leachate, the minerals (± *G. oxydans*) were rinsed with deionized water and dried at room temperature in a fume hood. The residual powder was then measured via XRD using the same analytical protocol outlined above for the pre-experiment characterization. A mortar and pestle were used to gently break apart any clumps of grains and the dark-colored crust that formed the top of leached minerals. Residues from sample days 0, 5, 10, and 15 were analyzed to track any mineralogical changes that occurred during leaching. Spectra were analyzed using MDI JADE software. The amount of amorphous SiO_2_ (Fig. 4b) was calculated using the degree of crystallinity (DOC) method^35,36^, where the total crystalline area of the spectra is divided by the sum of the crystalline and amorphous area. Previously, this method has been used to determine amorphous content of other XRD spectra at the Cornell Center for Materials Research and found to have an uncertainty of < 5%^70^.

High-resolution images of experimental residues (Fig. 1) were collected using a Jeol JSM-IT700HR scanning electron microscope (SEM). Prior to SEM imaging samples were air dried and then mounted on carbon tape. High resolution secondary electron images were collected under high vacuum mode using an accelerating voltage of 3 kV. Chemical maps (Fig. 1b-d, i-j) and compositional spectra were acquired using an Oxford Ultim Max energy dispersive x-ray spectroscopy (EDS) detector. EDS maps and spectra were collected in low vacuum mode (at 50 Pa) using an accelerating voltage of 20 kV.

Grains from the solid experimental residues were mounted on carbon tape and additional measurements were performed using a WITec Alpha300R Raman spectrometer in the Department of Earth and Atmospheric Science at Cornell University. Measurements were performed with a green 532.067 nm laser and utilizing a 100x objective lens. Analyses were conducted using a grating of 300 g/mm with a spectral center of 2300 cm^− 1^, laser power of 2 mW. Fluorescence often obscured any peaks, but when peaks were visible the elevated background from fluorescence was subtracted from the spectrum using the ParticleScout software.

Organic acids present in select samples were derivatized then identified via gas chromatography-mass spectrometry (GC-MS), and gluconic acid was quantified via high performance liquid chromatography (HPLC), both in triplicate. For GC-MS, 0.2 mL aliquots of leachate were pipetted into 2 mL vials and dried under vacuum overnight. The dewatered acids were derivatized using excess N, O-Bis(trimethylsilyl)trifluoroacetamide at 70 °C for 1 h before again being dried overnight. The derivatized acids were then reconstituted with 1 mL dichloromethane before analysis, using the same GC-MS system as prior work^71^. For HPLC, 0.1 mL leachate was pipetted into a glass HPLC vial and diluted with HPLC water to a 1 mL total volume. The system was calibrated to quantify gluconic acid, but was otherwise the same system as prior work^72^.

## Supplementary Information

Below is the link to the electronic supplementary material.

Supplementary Material 1

Supplementary Material 2

## Data Availability

The majority of data are available in the main text or the supplementary materials. XRD datasets generated during the current study are available in a Zenodo repository titled “XRD Raw data for SCO bioleaching experiments solid residues” which can be accessed here: 10.5281/zenodo.15658356.
