# Supplementary material for: Bioleaching of olivine and enstatite with formation of Mg-oxalate mediated by engineered Gluconobacter oxydans
Source: Sci Rep. 2026 Aug 5;16:25317. doi: 10.1038/s41598-026-63817-0 (PMC13473492; doi:10.1038/s41598-026-63817-0)
Supplement: Supplementary file 1 [file 41598_2026_63817_MOESM2_ESM.docx]

Supplementary Materials for

Bioleaching of olivine and formation of Mg-oxalate mediated by engineered biology

**Authors:** Jacob D. Klug^1^, Luke Plante^2^, James L. Adair^2^, Alia Almansoori^2^, Stephanie Murillo Maikut^1^_,_ Buz Barstow^2*^, Esteban Gazel^1*^

**Affiliations:**

^1^Department of Earth and Atmospheric Sciences, Cornell University, Ithaca, NY 14853

^2^Department of Biological and Environmental Engineering, Cornell University, Ithaca, NY 14853

***Corresponding authors:** Esteban Gazel: [egazel@cornell.edu](mailto:egazel@cornell.edu), Buz Barstow, [bmb35@cornell.edu](mailto:bmb35@cornell.edu)

**This file includes:**

Table S1

Figs. S1 to S2

**Other Supplementary Materials for this manuscript include the following:**

Data S1: Supplementary data (in separate excel file)

**Table S1.** San Carlos Olivine composition used for mass balance calculations. Composition represents average from measurements on commercially obtained olivine from ref [67].

**Fig. S1. Presence of Mg-oxalate confirmed with Raman spectroscopy.** Background subtracted Raman spectrum of glushinskite (MgC_2_O_4_ • 2H_2_O). Peak positions consistent with glushinskite are shown with a dashed line.

**Fig. S2. Experiment starting materials are well characterized and homogenous.** Images of starting geologic materials for bioleaching experiments with a pure San Carlos Olivine on the left and San Carlos Olivine + enstatite on the right.

**Fig. S3. XRD spectra from pure olivine experiments.** Experiments where *G. oxydans* was in direct contact with olivine are on the left, and those where it was absent are shown on the right.

**Fig. S4. XRD spectra from pure olivine + enstatite experiments.** Experiments where *G. oxydans* was in direct contact with olivine + enstatite are on the left, and those where it was absent are shown on the right.
